# Congenital hyperinsulinism in the Ukraine: a 10-year national study

**DOI:** 10.3389/fendo.2024.1497579

**Published:** 2024-12-17

**Authors:** Evgenia Globa, Henrik Thybo Christesen, Michael Bau Mortensen, Jayne A. L. Houghton, Anne Lerberg Nielsen, Sönke Detlefsen, Sarah E. Flanagan

**Affiliations:** ^1^ Ukrainian Scientific and Practical Center of Endocrine Surgery, Transplantation of Endocrine Organs and Tissues of MoH of Ukraine, Kyiv, Ukraine; ^2^ Hans Christian Andersen Children’s Hospital, Odense University Hospital, Odense, Denmark; ^3^ Department of Surgery, Upper GI and HPB Section, Odense University Hospital, Odense, Denmark; ^4^ The Genomics Laboratory, Royal Devon University Healthcare NHS Foundation Trust, Exeter, United Kingdom; ^5^ Department of Nuclear Medicine, Odense University Hospital, Odense, Denmark; ^6^ Department of Pathology, Odense University Hospital, Odense, Denmark; ^7^ Department of Clinical and Biomedical Science, University of Exeter Medical School, Exeter, United Kingdom

**Keywords:** congenital hyperinsulinism, hypoglycemia, genes, treatment, outcomes

## Abstract

**Introduction:**

Congenital Hyperinsulinism (CHI) has not been previously studied in Ukraine. We therefore aimed to elucidate the genetics, clinical phenotype, histological subtype, treatment and long-term outcomes of Ukrainian patients with CHI.

**Methods:**

Forty-one patients with CHI were recruited to the Ukrainian national registry between the years 2014-2023. Genetic testing (n=40), 18F-fluorodihydroxyphenylalanin and 68Ga-DOTANOC PET/CT imaging followed by surgical treatment and subsequent histological analysis (n=19) was performed through international collaboration.

**Results:**

Pathogenic variants were identified in 19/22 (86.3%) individuals with persistent CHI (p-CHI) and 8/18 (44.4%) with early remission CHI (er-CHI). Pathogenic variants in the K-ATP channel genes were the only identified genetic cause of p-CHI (*ABCC8* (n=17) and *KCNJ11* (n=2)) with greater genetic heterogeneity observed in those with er-CHI (*ABCC8* (n=3), *KMT2D* (Kabuki Syndrome, n=1), Beckwith-Wiedemann syndrome (n=2) and *INSR* (Donohue syndrome (n=2)). Histological analysis performed on 19 children with persistent CHI confirmed focal disease in 14 (73.7%), diffuse disease in two (10.5%) and atypical histology in three (15.8%). After surgery, complete recovery was observed in all 14 with focal disease, while relapse occurred in three patients with diffuse or atypical histology.

**Conclusion:**

A genetic diagnosis was achieved for 67.5% (27/40) of the cohort with a higher pick-up rate observed in those with p-CHI. The genetics and imaging studies enabled subtype-targeted treatment with surgical cure achieved in all individuals with focal disease.

## Introduction

Congenital hyperinsulinism (CHI) is a clinically, genetically and histologically heterogeneous condition caused by inappropriate insulin secretion during hypoglycemia. Variants in over 30 genes are known to cause CHI which can present in isolation or as part of a syndrome (1). Persistent CHI (p-CHI) caused by loss-of-function variants in the ATP-sensitive potassium (KATP) channel genes *KCNJ11* and *ABCC8*, account for 40–50% of cases (2). In contrast, a genetic cause of disease is not identified in the majority of individuals with transient CHI (2).

Histologically, CHI can be classified as diffuse, focal or atypical disease (3). Atypical CHI is an umbrella term for several different histologies (4). Although the clinical presentation can be similar between histological subgroups, the molecular mechanisms, mode of inheritance, and treatment modalities are different. For all patients the cornerstone of clinical management is a prompt diagnosis and early initiation of appropriate treatment since there is a high risk of neurological complications associated with all forms of CHI (5–9).

Management of hypoglycemia includes pharmacotherapy and pancreatic surgery in case of drug unresponsiveness or focal disease (10, 11). Focal CHI is caused by a lesion limited to a specific region of the pancreas. These lesions develop when a recessive KATP channel pathogenic variant on the paternal chromosome is unmasked by uniparental isodisomy within the pancreatic tissue (12). Preoperative localization is important to identify the suspected lesion(s) as a limited (partial) pancreatectomy/focal excision can be a curable tool with low risk of long-term complications (3, 10, 11). Also, intraoperative frozen section analysis helps to identify the focal lesion and to remove it with free surgical margins. Individuals with a medically-unresponsive diffuse disease may require near total pancreatectomy with unfavorable long-term outcomes, including a high risk of residual hypoglycemia, diabetes mellitus and exocrine pancreatic insufficiency (13, 14).

An 18F–dihydroxyphenylalanine (18F-DOPA) PET-CT scan is recommended for the preoperative localization of focal lesions (15–17) and has fundamentally changed management strategies for CHI. Particularly for patients with focal CHI, 18F-DOPA PET-CT scanning has shown superiority over 68Ga-1,4,7,10-tetraazacyclododecane-1,4,7,10-tetraacetid-acid-1-NaI3-octreotide (68Ga-DOTANOC) PET/CT in preoperative prediction of a focal lesion (18). It also allows differentiation of focal and diffuse forms with a high sensitivity (88%) and specificity (94%) (15, 16, 19), with an accuracy of 88-100% (16, 17, 20–22). Pancreatic surgery should be guided by preoperative imaging and subsequently by intraoperative visual inspection, palpation, ultrasound, frozen section analysis (if needed using piecemeal resection until the focus is identified in complicated cases) and final pathological diagnosis based on microscopy of the entire resection specimen (10, 23–25).

International guidelines have recently been devised to aid in the diagnosis and medical management of CHI which requires a multidisciplinary team including an endocrinologist, radiologist, surgeon and surgical pathologist. These guidelines provide recommendations for rapid genetic testing, advanced radiological imaging and the use of mainstay drugs, such as diazoxide (26).

Given the rarity and complexity of this disease, we investigated the incidence, genetics, clinical phenotype, histological type, treatment modalities and outcomes in patients with CHI living in Ukraine.

## Materials and methods

### Subjects

All individuals presenting with CHI between 1^st^ January 2014 and 31^st^ of December 2023 were recruited to the national CHI registry. These individuals had been referred to the tertiary Endocrine Center in Kyiv from across Ukraine because of persistent or recurrent hypoglycemia not explained by maternal diabetes or medication, intrauterine growth retardation or asphyxia. At referral, all individuals required frequent feeding and/or drug therapy. The diagnosis of CHI was made on the basis of increased insulin action and/or inadequate suppression of plasma insulin during either spontaneous or fasting-induced hypoglycemia that was diagnosed within the first 12 months of life (26). Individuals with known or suspected metabolic causes of hypoglycemia (e.g. congenital disorders of glycosylation, glycogen storage disorders) were excluded.

Patients were classified as having early remission CHI (er-CHI) if spontaneous remission was observed by the age of two years with no evidence of hypoglycemia within a period of 24 months. Individuals were classified as having p-CHI when the hypoglycemia persisted beyond the age of two years and required constant glucose monitoring, prolonged dietary carbohydrate supplementation and medical treatment or pancreatic surgery before the age of 2 years.

The study complied with the Declaration of Helsinki with informed consent obtained from all patient’s parents. Ethical approval was received from the Genetic Beta-cell Research Bank for the genetic testing performed in Exeter (517/WA/0327).

### Genetic testing

Leukocyte DNA was available for genetic testing on 40 of the 41 probands (97.5%). Genetic testing was undertaken at the Exeter NHS Genomics Laboratory UK (n=39) or via a commercial laboratory (n=1; Centogene, Rostoсk, Germany). The screening approach differed between individuals because of changes in genetic technology and an improved knowledge of the genetics of CHI over the 10-year period.

For patients tested in Exeter, Sanger sequencing of the *ABCC8* and *KCNJ11* genes was performed as a first line test in 32 individuals using standard methods. Seven individuals also underwent Sanger sequencing of *HK1* (n=1), *GLUD1* and *HK1* (n=1), or *HNF4A* (n=5). To search for deletions in the *HK1* non-coding regulatory region, digital droplet PCR (ddPCR) was undertaken in the two patients who had undergone *HK1* Sanger sequencing, as previously described (27). For eight patients, targeted next-generation-sequencing (tNGS) was undertaken to search for single nucleotide variants and copy number variants in a minimum of 12 genes (*ABCC8*, *CACNA1D*, *GCK*, *GLUD1*, *HADH*, *HNF1A*, *HNF4A*, *INSR*, *KCNJ11*, *PMM2*, *SLC16A1* and *TRMT10A*) (28). For five patients, the *KMT2D*, *KDM6A* and *HK1* genes were also analyzed by tNGS. Methylation-specific multiplex ligation-dependent probe amplification (MS-MLPA) (n=2) was performed (MRC-Holland kit ME030-C3) in two patients with a clinical suspicion of Beckwith-Wiedemann syndrome, with exome sequencing performed in Germany on one further patient with syndromic disease.

The American College of Human Genetics Guidelines was used to classify variants (29). Parents of a proband with a variant in one of the analyzed genes were tested by Sanger sequencing for the respective variant.

### PET/CT-based assessment

Since 18F-DOPA/68Ga PET-CT is not currently available in Ukraine, pancreatic imaging was performed through an international collaboration with the hyperinsulinism center in Hans Christian Andersen Children’s Hospital and Odense University Hospital in Denmark (n=18) and the DTZ in Berlin, Germany (n=1). PET-CT scans were acquired on a GEDiscovery PET/CT scanner (GE Medical System, Waukesha, WI, USA) and analyzed on a Dexus AW server 2.0. 18F–DOPA was produced by the electrophilic method. The patients were injected with 18F–DOPA (n=19) and 68Ga-DOTANOC (n=3) 4 MBq/kg, minimum 30 MBq preoperatively as previously described (18).

### Surgery and histological analysis

Pancreatic surgery and subsequent histological analysis of the resected tissue was performed at Odense University Hospital (OUH) in Denmark (n=18) or in the surgical department of Bethel clinic, Bielefeld, Germany (n=1). At OUH, pancreatic tissue histology was assessed by intraoperative frozen section analysis, using hematoxylin-eosin staining and in most cases with the addition of tolouidine blue staining and/or immunohistochemistry for synaptophysin and insulin in difficult cases. After surgery, the tissues were analyzed by microscopy of 4 µm thick sections stained with hematoxylin-eosin and cut from formalin fixed, paraffin embedded specimens. If necessary, immunohistochemical staining for markers such as synaptophysin, insulin, glucagon, somatostatin and p57 was performed.

### Statistical analysis

Clinical characteristics are presented as median (interquartile range). Birth weight Z-scores were calculated using WHO standards, accessed through the Zanthro package in STATA 16 (StataCorp, Texas, USA). Mann-Whitney U-test and Fisher’s exact test were used for comparative statistics. Significant level was set as p<0,05.

## Results

We recruited 41 cases (56% female) with CHI diagnosed before 12 months of age to the CHI registry. All 41 probands originated from Ukraine and did not have a family history of consanguinity or diabetes/HI in first degree relatives. Limited data was available for one patient who was lost to follow up and this individual was therefore not included in the clinical descriptions of the Ukrainian cohort.

**Figure 1:**
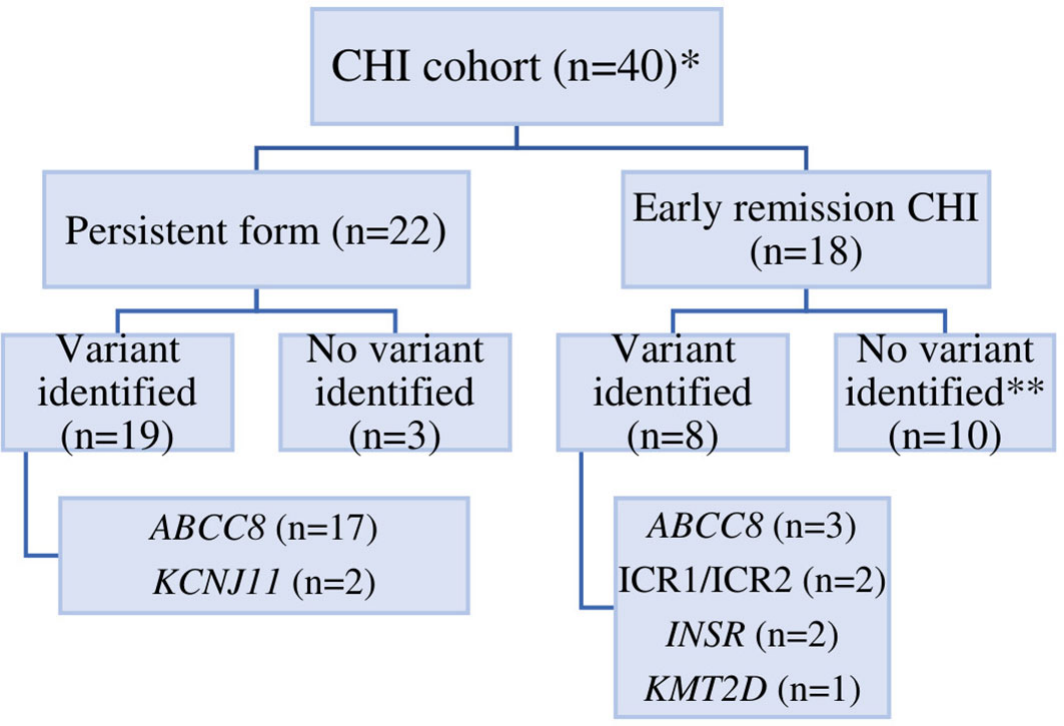
The distribution of CHI in Ukraine according to genetic etiology. An * indicates that 1 patient was lost to follow up, ** an *ABCC8* p.(Arg495Gln)/p.(Cys1491Arg) VUS was identified in one individual.

Of the 40 individuals, 18 (45%) had er-CHI and 22 (55%) had p-CHI (**Figure 1**). Within the er-CHI group, five individuals (12.5%) had syndromic disease. Of these, two had died within infancy. No differences (p>0.05) in age at diagnosis, sex, glucose concentration at presentation, birth weight SDS, C-peptide and insulin levels were observed between individuals within the p-CHI and er-CHI groups (**Table 1**).

**Table 1:** Clinical characteristics of patients with CHI.

	Age at diagnosis of CHI (days)	Birth weight, SDS	Glucose at presentation (mmol/l)	C-peptide (ng/ml)	Insulin (mU/L)	Male/ Female (%)
p-CHI (n=22)	3.5 [1.0:52.0]	0.9 [0.4; 2.1]	1.0 [0.5:1.2]	3.8 [1.4:8.0]	26.7 [10.7:43.6]	36.4/63.6
er-CHI (n=18)	17.0 [2.0:60.0]	0.2 [-0.5; 1.3]	1.3 [0.6:2.5]	2.7 [0.9:4.5]	13.9 [6.6:31.6]	55.6/44.4

p-CHI, persistent CHI; er-CHI, early remission CHI. Data presented as median results and [IQR], p>0,05.

### Medical management of CHI

Octreotide (5-25 mcg/kg per day) was the first line of continuous treatment in 24 cases since diazoxide is not easily accessible in Ukraine. In six octreotide-unresponsive patients, diazoxide was outsourced and given as a second line treatment (5-20 mg/kg per day). In three severe cases, glucocorticoids (GC) were also used. The treatment regimen was guided by drug availability, drug responsiveness and knowledge of treatment modalities for CHI at the time. Glucagon injections had routinely been given in case of severe hypoglycemia. Nineteen individuals with severe p-CHI underwent PET-CT scanning followed by surgical treatment. The median age of surgical treatment was 6 months [IQR: 4,0; 8,5 months] and age at last follow up was 6 years [IQR: 1,1; 9,5 years].

### Genetic findings

The genetic cause of CHI was determined in 27/40 (67.5%) individuals (*ABCC8* n=20, *KCNJ11* n=2, *INSR* n=2, *KMT2D* n=1, chr11p15 imprinting defects n=2). An *ABCC8* variant of uncertain clinical significance (VUS) was identified in one individual. Overall, a higher pickup rate was observed in p-CHI compared to the er-CHI group (19/22 (86.4%) vs 8/18 (44.4%); p=0.007).

### Genetics and medical management of the p-CHI cohort

In p-CHI, all 19 individuals with a genetic diagnosis had variants in the KATP channel genes (*ABCC8* (n=17), *KCNJ11* (n=2)), representing the only cause of p-CHI in Ukraine. Fourteen had a heterozygous, paternally inherited variant, four were compound heterozygous and one individual (Patient 8) had a dominant variant that had arisen *de novo* (**Table 2**).

**Table 2:** Genetic causes, inheritance, histological forms and treatment in patients with genetic findings or/and p-CHI from the Ukrainian CHI Registry.

Patient	Gene	Variant Protein Description	Variant DNA Description	Parental genotypes		Pathogenicity	CHI form	Treatment	Histological form	PET CT/form/location
				Mother	Father					
1.	*ABCC8*	p.(Gln444His)	c.1332G>T	WT	p.(Gln444His)	P	p-CHI	Surgical: A focal lesion in the pancreatic head required a duodenum-preserving head resection and Roux-en-Y pancreatico-jejunostomy to ensure complete resection of the focal lesion Outcome: resolved	Focal	18F-DOPA/Focal/ pancreatic head
2	*ABCC8*	p.?	c.4415-13G>A	WT	p.?	P	p-CHI	Surgical: Enucleation with preservation of the pancreatic duct Outcome: resolved	Focal	18F-DOPA/Focal/ pancreatic body, in processus uncinatus vеrу closely to the distal рагt of second part of duodenum
3	*ABCC8*	p.(Gln444His)/ p.(Gln923Ter)	c.1332G>T/ c.2767C>T	p.(Gln923Ter)	p.(Gln444His)	P/P	p-CHI	Medications GC+O+D. Lost to follow up at 2 y	NA	NA
4	*ABCC8*	p.(Arg1437Ter)	c.4309C>T	WT	p.(Arg1437Ter)	P	p-CHI	Surgical: Focal resection Outcome: resolved	Focal	18F-DOPA/Focal/ pancreatic head
5	*ABCC8*	p.(Arg1251Ter)/p.(Tyr1287Ter)	c.3751C>T/ c.3861C>A	p.(Tyr1287Ter)	p.(Arg1251Ter)	P/P	p-CHI	Surgical: 90% pancreatectomy Outcome: resolved	Diffuse	68Ga-DOTANOC and 18F-DOPA/Diffuse
6	*ABCC8*	p.(Gln923Ter)	c.2767C>T	–	p.(Gln923Ter)	P	p-CHI	Surgical: Focal resection Outcome: resolved	Focal	18F-DOPA/Focal/ Body-tail
7	*ABCC8*	p.(Trp75Ter)/ p.(Gln444His)	c.225G>A/ c.1332G>T	p.(Gln444His)	p.(Trp75Ter)	P/P	p-CHI	Medications: O Age at last follow-up 8y	NA	NA
8	*ABCC8*	p.(Asp1506Glu) *de novo* dominant variant	c.4518C>G	WT	WT	P	p-CHI	Surgical: 80% pancreatic resection Outcome: Relapse, is being treated with LASA, age at last follow-up 7y	Diffuse	18F-DOPA/Diffuse
9	*ABCC8*	p.(Gly827fs)	c.2480del	WT	p.(Gly827fs)	P	p-CHI	Surgical: Enucleation with preservation of the pancreatic duct Outcome: resolved	Focal	18F-DOPA/Focal/ Pancreatic body
10	*ABCC8*	p.?	c.3992‐9G>A	WT	p.?	P	p-CHI	Surgical: Resection of lesion Outcome: resolved	Focal	18F-DOPA/Focal/Pancreatic body
11	*ABCC8*	p.(Arg1394Leu)	c.4181G>T	WT	p.(Arg1394Leu)	LPP	p-CHI	Surgical: Focal resection Outcome: resolved	Focal	18F-DOPA/Focal/pancreatic head
12	*ABCC8*	p.(Arg1539Ter)	c.4615C>T	WT	p.(Arg1539Ter)	LP	p-CHI	Surgical: Left-sided pancreatectomy due to a large focal lesion in the tail of the pancreas Outcome: resolved	Focal	18F-DOPA/Focal/Large lesion in distal and central left pancreas
13	*ABCC8*	p.(Arg934Ter)	c.2800C>T	WT	p.(Arg934Ter)	P	p-CHI	Surgical: Focal resection Outcome: resolved	Focal	18F-DOPA/Focal/at the border between the pancreatic body and tail
14	*ABCC8*	p.?	c.3560 + 1G>A	WT	p.?	P	p-CHI	Surgical: Focal resection Outcome: resolved	Focal	18F-DOPA/Focal/ pancreatic head
15	*ABCC8*	p.(Arg1539Ter)	c.4615C>T	WT	p.(Arg1539Ter)	LP	p-CHI	Surgical: Focal resection, the suspected ectopic pancreatic tissue was identified in the mesentery adjacent to the ilium and resected Outcome: resolved	Focal	18F-DOPA/Focal/ Pancreatic body. A smaller area resembling normal ectopic pancreatic tissue was detected close to mesenterial vessels, inferior to the uncinate process
16	*ABCC8*	(p.Arg1393Leu)/(p.Gln444His)	c.4178G>T/c.1332G>T	(p.Arg1393Leu)	(p.Gln444His)	P/P	p-CHI	Medications: LASA	NA	NA
17	*ABCC8*	p.(Tyr179Ter)	c.536_539del	WT	Not tested	P	p-CHI	Surgical: Initial focal resection of the pancreatic head and subsequent duodenal-preserving re-resection proved insufficient. Therefore, the patient ultimately needed a complete Whipple’s procedure to achieve normoglycemia by fasting test Outcome: resolved	Focal	18F-DOPA/Focal/ large (21x11 mm) focal lesion with ill-defined borders in the pancreatic head
18	*KCNJ11*	p.(Phe333Ser)	c.998T>C	WT	p.(Phe333Ser)	P	p-CHI	Surgical: During surgery, the main pancreatic duct could not be preserved, necessitating division of the gland and a pancreatico-gastrostomy Outcome: resolved	Focal	68Ga-DOTANOC and 18F-DOPA/Focal/Focus between the head and body of the pancreas
19	*KCNJ11*	p.(Ile210Thr)	c.629T>C	WT	p.(Ile210Thr)	LP	p-CHI	Surgical: Focal resection Outcome: resolved	Focal	18F-DOPA/Focal/ pancreatic head to uncinated process
20	*ABCC8*	p.(Gly827fs)	c.2480del	WT	p.(Gly827fs)	P	er-CHI	Medications/Meal	NA	NA
21	*ABCC8*	p.(Arg705Ter)	c.2113C>T	WT	p.(Arg705Ter)	P	er-CHI	Medications/Meal	NA	NA
22	*ABCC8*	p.(Arg495Gln)/ p.(Cys1491Arg)	c.1484G>A/ c.4471T>C	p.(Cys1491Arg)	p.(Arg495Gln)	VUS	er-CHI	Medications/Meal	NA	NA
23	*ABCC8*	p.(Tyr179Ter)	c.536_539del	WT	p.(Tyr179Ter)	P	er-CHI	Medications/Meal	NA	NA
24	*KMT2D*	p.(His2951fs)	c. 8852del	NA	NA	LP	er-CHI	Medications/Meal	NA	NA
25*	*INSR*	p.(Tyr94Ter)/ p.(Arg1020Ter)	c.282T>G/ c.3058C>T	p.(Arg1020Ter)	*de novo*	P/P	er-CHI	Medications/Meal	NA	NA
26	*INSR*	p.(Cys35Tyr)/p.(Thr940Pro)	c.104G>A/c.2818A>C	p.(Thr940Pro)	p.(Cys35Tyr)	LP/LP	er-CHI	Medications/Meal, metformin	NA	NA
27	chr11p15 imprinting defect	IC2 hypomethylation and IC1 hypermethylation	NA	NA	NA	NA	er-CHI	Medications/Meal	NA	NA
28	chr11p15 imprinting defect	IC2 hypomethylation and IC1 hypermethylation	NA	NA	NA	NA	er-CHI	Medications/Meal	NA	NA
29	No variant	NA	NA	NA	NA	NA	p-CHI	Surgical: 66% resection of pancreatic preserving the pancreatic head Outcome: Resolved	Atypical	18F-DOPA/Atypical/ Atypical changes in enlarged pancreatic tail
30	No variant	NA	NA	NA	NA	NA	p-CHI	Surgical: 80% pancreatic resection Outcome: Relapse, treatment with LASA till 10 y.o.	Atypical	68Ga-DOTANOC and 18F-DOPA/Atypical
31	No variant	NA	NA	NA	NA	NA	p-CHI	Surgical: Left-sided 80-85% pancreatectomy Outcome: Relapse, is being treated with D+LASA+GC, age at last follow-up 5.5 y	Atypical	18F-DOPA/Diffuse, with atypical area in the head of the pancreas

NA, Data not available; p-CHI, persistent CHI; er-CHI, early remission CHI; Variants were classified according to ACMG guidelines. P, Pathogenic; LP, Likely Pathogenic; WT, wild type genotype; VUS, Variant of Uncertain Significance.

Treatment: O, octreotide; LASA, long-acting somatostatin analogues; D, diazoxide; GC, glucocorticoids.

*This patient has been previously reported doi: 10.1111/dme.15013.

All 22 cases with p-CHI had a difficult unstable and poorly controlled course of the disease with some individuals requiring treatment with a combination of therapies. Among the 22 individuals, 11 (50%) had epilepsy and/or psychomotor retardation, with five being treated with antiepileptic drugs at the time of referral. Neurological follow-up data were not recorded in the database and would require a prospective study.

Nineteen individuals with poor response to treatment underwent 18F-DOPA PET-CT scanning (3 additionally with 68Ga) and received surgical treatment (**Figure 1**; **Table 2**). Based on the histological findings, 14 patients (73.7%) were classified as having focal KATP channel CHI, two (10.5%) diffuse KATP channel CHI, and three patients had an atypical histological form of CHI (15.8%) (**Table 2**). 18F-DOPA PET/CT predicted the histological type in all cases, including the three histologically atypical patients (Patients 29-31), who presented with irregular labeling not typical for either KATP channel diffuse or focal CHI; and the Patient 12 and 17 with unusually large focal lesions.

Of the two patients with diffuse CHI, one had a dominant *ABCC8* variant, and the other was compound heterozygous for two *ABCC8* variants. A paternally inherited variant in *ABCC8* (n=11) or *KCNJ11* (n=2) was identified in 13 individuals with focal disease. In the remaining patient the *ABCC8* variant was presumed paternal (Patient 17, **Table 2**). For the three patients with atypical histology, comprehensive genetic testing by tNGS (n=3) did not identify a pathogenic variant in a known CHI gene.

**Figure 2:**
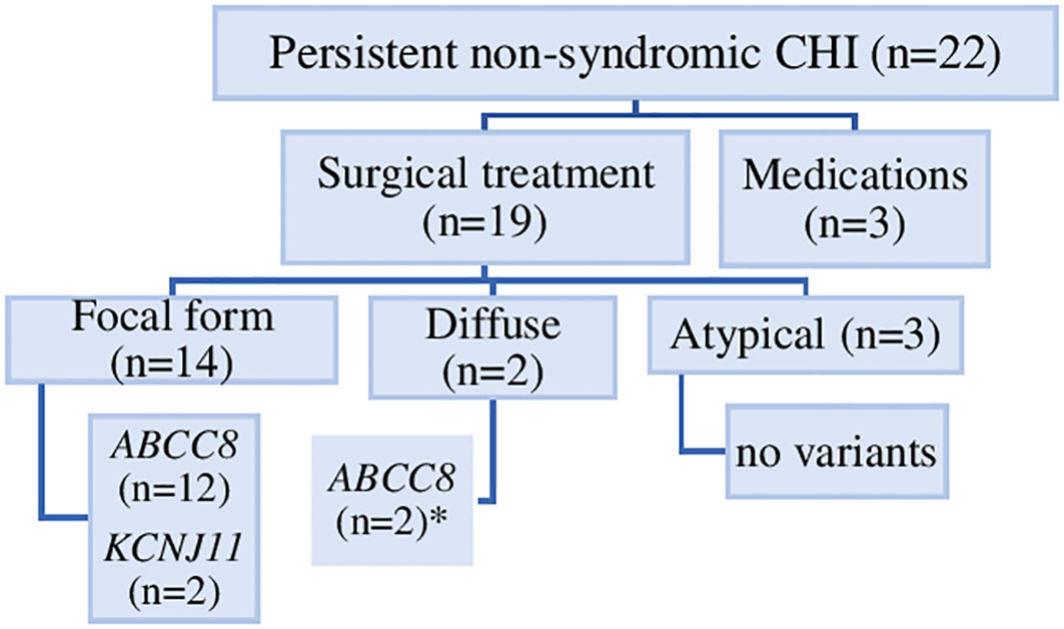
Genetic causes, histological forms and treatment of 22 individuals with persistent non-syndromic CHI. Diffuse pancreatic disease was identified in two individuals (*), one with compound heterozygous *ABCC8* variant and one with a dominantly-acting *ABCC8* variant.

Of the 19 patients who underwent surgery, 14 had a focal lesion confirmed by freeze microscopy and with focal adenomatous hyperplasia in the final histology report. Of these, ten had a surgical enucleation of the focal lesion. In the remaining four patients, larger pancreatic resections were necessary depending on technical/anatomical reasons. Five patients with diffuse/atypical CHI underwent 66-90% pancreatic resection. Further details regarding the surgical treatment and histological findings for each patient are provided in the **Table 2** and **Figure 2**.

Postoperative complications were relatively rare and included transient fasting hyperglycemia and transient exocrine insufficiency (up to 3 months) (n=1), and postoperative hernia (n=1), both observed in patients with focal CHI. Complete hypoglycemia recovery was observed in all patients with a focal lesion with a normal postoperative fasting test and normal glucometer checks at follow-up.

Among the two patients with diffuse CHI, one with a dominant *de novo ABCC8* variant developed a relapse of the disease after an 80% pancreatic resection. This patient received treatment with a long-acting somatostatin analogue (LASA), which prevented further severe hypoglycemia. In one case with compound heterozygous *ABCC8* variants, a 90% pancreatic resection was curative. Histologically, in both cases, giant cell nuclei in some beta cells in islets of Langerhans was found.

Relapse of CHI occurred in 2/3 individuals with atypical pancreatic disease. For one, LASA was initiated after the surgery and was stopped 9 years later. The second patient continued to take octreotide, diazoxide and GC following surgery, with GC stopped 1-year post-surgery (**Table 2**). This individual continues to have severe hypoglycemic comas at the age of 5.5 years, however these occur with less frequency (about once a year). The diagnosis of CHI in the 3 cases with atypical disease was relatively late (87^th^, 273^th^ and 330^th^ day of life). The latter two patients had presented to neurologists before the age of 3 months with seizures and epilepsy, however hypoglycemia was not confirmed at this time. Histologically atypical CHI was confirmed (**Table 2**).

### Genetics and clinical features in er-CHI

A pathogenic variant was identified in 8/18 (44.4%) patients with er-CHI (*ABCC8* (n=3), *KMT2D* (n=1), aberrant methylation at the chr11p15.5 DMR (n=2), and *INSR* (n=2)). In one additional patient a VUS in *ABCC8* was identified (**Table 2**, **Figure 1**).

The three children with *ABCC8* variants had inherited a recessively acting null variant from their unaffected father. The CHI was diagnosed early in all (<25^th^ day of life) and had been treated with carbohydrate supplementation feeding only. An 18F-DOPA PET-CT scan was not performed in any of these cases and therefore a diagnosis of focal disease was not investigated.

The finding of a *KMT2D* variant confirmed Kabuki syndrome in one individual. This patient was born prematurely with the pregnancy complicated by polyhydramnios. The child had specific facial features, oral cleft, ataxia, muscular hypotonia, dystonia, motor delay, delayed speech, and language development, hydronephrosis and gastrointestinal anomalies which were consistent with the genetic diagnosis.

In two individuals’ aberrant methylation at Chr11p15.5 confirmed Beckwith Wiedemann syndrome. One of these children had undergone four operations for severe macroglossia and later developed nephroblastoma at the age of 5 years.

The two patients with compound heterozygous *INSR* variants had a birth weight of -2.9 and -4.3 SDS and additional features consistent with the genetic diagnosis of Donohue syndrome. One had intermittent transient hypoglycemia alternating with hyperglycemia. In the second case hypoglycemia was mild and metformin was prescribed due to hyperinsulinemia (3897 mIU/mL, C-peptide 77.8 ng/ml (normal range 1.1-4.4)); she had giant ovarian cysts (>7 cm) and intestinal compression, which required ovariectomy. Both children died before the age of 4 months.

One of the 10 children without a genetic diagnosis had a relapse of HI at the age of 4y 6 mo. From that age, he experienced episodic hypoglycemic comas with increasing frequency and severity. An 18 F-DOPA PET-CT scan did not detect an abnormality and LASA treatment was initiated at the age of 7 years with satisfactory outcome.

## Discussion

In this first report of children with CHI in Ukraine, we recruited 41 cases (56% female) with CHI and studied clinical, paraclinical and genetics which allowed for subtype-specific treatment through international collaboration.

A genetic diagnosis was confirmed in 67.5% of our cohort, with the highest pick-up rate observed amongst those with p-CHI (86.4%). This is similar to that reported in the Japanese population (30), but higher compared to other European studies (2, 31–36). Disease-causing variants in the KATP-channel genes were most common (n=22/40) and the only cause of p-CHI and non-syndromic er-CHI in our cohort. This differs from other countries where greater diversity in the genetic causes of CHI has been reported (32–37). Notably, no patients were identified with the otherwise relatively frequent activating *GCK* or *GLUD1* mutations.

Paternally inherited KATP-channel variants were most common (73.7%) in our cohort similar to other studies (2, 30, 32, 35, 36, 38) and no homozygous variants were identified which may be explained by the absence of consanguinity in our cohort. Of note, the *ABCC8* variant p.(Gln444His) was seen in four unrelated probands. Interestingly, all four individuals were referred from northwestern regions of Ukraine raising the possibility of a founder variant (**Table 2**). Given however that the p.(Gln444His) variant has been frequently reported in other European and US CHI cohorts (24, 39) it is also possible that this is just a chance finding with haplotype analysis being required to investigate this further.

In the er-CHI group, rare syndromic forms of hyperinsulinism were most common (5/18; 27.8%). These included Donohue syndrome, Beckwith–Wiedemann syndrome and Kabuki syndrome. The two patients with Donohue syndrome experienced a transient period of hypoglycemia and died at an early age, as has been described elsewhere. Three patients with isolated er-CHI (16,7%) had a paternally inherited recessive *ABCC8* variant which was unexpected given that these variants are most commonly associated with focal p-CHI (2, 34). A small number of individuals with er-CHI and a paternal *ABCC8* variant have however been described (30, 36). As our three patients did not undergo imaging studies or surgery, a mild form of focal disease with spontaneous remission could not be ruled out. It also remains possible that the *ABCC8* variants were not causative of their disease (40) and that they were incidental carriers of *ABCC8*-HI, or there is a second disease-causing variant in the germline or a mosaic somatic variant that was not detected on routine screening. Since patients with hypoglycemia not explained by maternal diabetes or medication, intrauterine growth retardation or asphyxia were not routinely included in this study, we were unable to estimate the prevalence of transient CHI in Ukraine.

For 19 patients who had undergone pancreatic surgery, genetic testing and imaging correctly predicted the focal vs diffuse histology. Patients with a paternally inherited *ABCC8/KCNJ11* variant causing focal disease had the most favorable outcome with complete recovery in the absence of significant postoperative complications or recurrence. Unfavorable outcomes in terms of cure were observed in patients without a genetic diagnosis and atypical histological disease. For two of these patients CHI relapsed after surgery, although the severity of hypoglycemia became more manageable with further drug treatment. Moving forward these patients should be prioritized for genetic discovery studies to help gain insights into the genetic mechanisms of atypical pancreatic disease with the aim to help improve medical management.

Notably, the average age of surgery was later in our cohort compared to Demirbilek study (41). This may be owing to the significant financial burden placed on families if they should source funding for 18F-DOPA PET-CT scan and surgery to be performed privately outside of Ukraine. Whilst we were able to form close international collaborations with European CHI centers specializing in free rapid genetic diagnostics, 18F-DOPA PET-CT scanning, surgical treatment and histological analysis, these data serve to reflect the inequities that still exist in healthcare provision around the world, which adds to the many challenges faced by the families and clinicians who are managing this complex condition (42). At the same time this implementation of rapid international collaboration between teams of geneticists, radiologists and surgeons has improved disease management in patients with p-CHI (especially those with the focal forms), with predicted better neurological outcomes and less financial and psychological burden on family members due to complete cure of the disease or alleviation of its symptoms.

In conclusion, we present the first report describing CHI in Ukraine highlighting the importance of a multidisciplinary team to improve treatment outcomes. Within the Ukrainian population, KATP channel variants were most common, accounting for all genetic diagnoses of p-CHI and non-syndromic er-CHI. The genetics and imaging studies enabled subtype-targeted treatment with surgical cure achieved in all individuals with focal p-CHI.

## Data Availability

The sequencing datasets presented in this article are not readily available because ethical approval only allows for the sharing of data through collaboration to experienced teams working on approved studies examining the mechanisms, cause, diagnosis and treatment of diabetes and other beta cell disorders. Requests to access the datasets should be directed to Prof S. Flanagan (s.flanagan@exeter.ac.uk).
